# Waist‐to‐height ratio for the prediction of gallstone disease among different obesity indicators

**DOI:** 10.1002/osp4.650

**Published:** 2023-01-12

**Authors:** Tien‐Shin Chou, Chih‐Lang Lin, Li‐Wei Chen, Ching‐Chih Hu, Jia‐Jang Chang, Cho‐Li Yen, Shuo‐Wei Chen, Ching‐Jung Liu, Cheng‐Hung Chien

**Affiliations:** ^1^ Division of Gastroenterology Department of Internal Medicine Keelung Chang Gung Memorial Hospital Keelung Taiwan, ROC; ^2^ Liver Research Unit Keelung Chang Gung Memorial Hospital Keelung Taiwan, ROC; ^3^ Community Medicine Research Center Keelung Chang Gung Memorial Hospital Keelung Taiwan, ROC

**Keywords:** central obesity, community health, gallbladder, insulin resistance, metabolic syndrome

## Abstract

**Background:**

Factors of metabolic syndrome such as obesity are well‐known risk factors for gallstone disease (GSD). There are different indicators of obesity, including weight, body mass index, waist circumference, and waist‐to‐height ratio. The predictive ability of different obesity indicators for GSD remains unclear.

**Objective:**

To explore the most efficient predictor of GSD among the different anthropometric indicators of obesity.

**Methods:**

This population‐based cross‐sectional study included 2263 participants who completed a questionnaire detailing their demographics, medical history, and lifestyle between 2014 and 2017 in Taiwan. Blood samples were collected and physical examinations, including anthropometric measurements, were performed. Gallstone disease was ascertained using ultrasonography. Multivariate analyses were performed to identify independent risk factors for GSD.

**Results:**

The overall prevalence of GSD was 8.8%. According to the multivariate analysis, individuals with a waist‐to‐height ratio ≥0.5 (odds ratio|odds ratios (OR) = 1.65, 95% confidence interval (CI) = 1.10−2.48, *p* = 0.017) had an increased risk of GSD. Diabetes was the main risk factor for GSD in men (OR = 2.06, 95% CI = 1.17−3.65, *p* = 0.013). Among women, waist‐to‐height ratio >0.5 (OR = 1.76, 95% CI = 1.03−3.02, *p* = 0.040) and current hormone drug use (OR = 2.73, 95% CI = 1.09−6.84, *p* = 0.033) were significant risk factors for gallstones.

**Conclusion:**

GSD was independently associated with central obesity and exogenous hormone intake in women. Among the anthropometric indicators used to assess central obesity, waist‐to‐height ratio was the most accurate predictor of GSD.

## Graphical abstract

Obesity is a risk factor of gallstone disease. Among anthropometric indicators of obesity, waist–to–height ratio has the best predictability of gallstone disease in women.

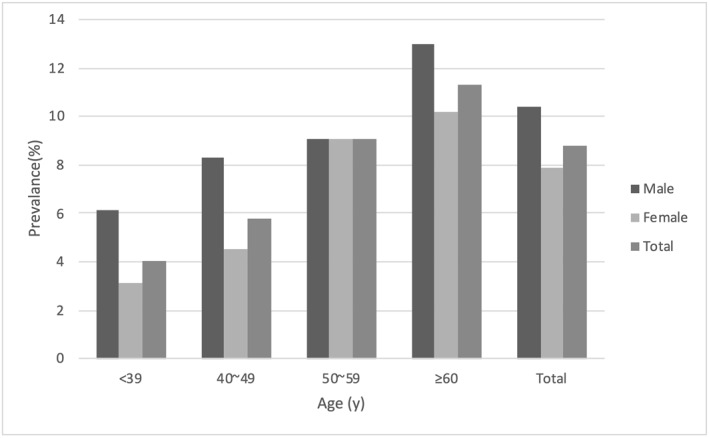

## 1 INTRODUCTION

Gallstone disease (GSD) is a highly common digestive disorder of multifactorial origin. Most patients with GSD are asymptomatic and are unaware of gallstones. Patients with symptomatic GSD and related complications require surgical removal of the gallbladder, which constitutes a strain on medical resources.^1^ Several epidemiological studies have indicated that age, sex, coffee consumption, tea consumption, physical inactivity, and most of the key components of metabolic syndrome, including central obesity, dyslipidemia, type 2 diabetes, and cirrhosis, are associated with GSD.^2^, ^3^ Understanding the epidemiology of GSD and its risk factors is important for identifying patients with gallstones and preventing their formation. Furthermore, a simple method in clinical practice to increase the diagnostic accuracy of GSD is crucial in assessing patients with symptoms or signs that may be related to complications of GSD, such as cholangitis.

Two nationwide studies conducted in the 1990s and 2000s indicated a marked increase in the prevalence of metabolic syndrome and obesity in Taiwan within a decade.^4^ Obesity, a factor in metabolic syndrome, is a well‐known risk factor for GSD. The association between obesity and GSD is attributable to increased hepatic secretion of cholesterol and impaired gallbladder motility.^5^ There are different indicators used in clinical practice to assess obesity, including weight, body mass index (BMI), waist circumference (WC), waist‐to‐hip ratio (WHR), and waist‐to‐height ratio (WHtR).

Gallstone disease is regarded as a disease that is strongly associated with atherosclerosis and cardiovascular disease,^6^ and a simple and accurate method to assess its risk factors is important in clinical practice and patient education. WHtR has been strongly associated with several chronic diseases, such as hypertension, cardiovascular events, type 2 diabetes, and metabolic syndrome, and has thus been extensively researched.^7^ Several underlying mechanisms support the superiority of WHtR over other anthropometric indicators in the assessment of obesity.^8^, ^9^, ^10^, ^11^ Nevertheless, the predictability of different indicators of obesity for GSD, including the WHtR, is still unclear.

In addition to obesity, previous studies have investigated the association between GSD and other factors of metabolic syndrome, such as hypertension, hyperglycemia, and dyslipidemia. However, these studies reported inconsistent and inconclusive results.^1^, ^12^ Exogenous hormone intake and parity are considered risk factors for GSD, but the role of sex differences in the development of GSD remains unclear.^12^, ^13^, ^14^

This community‐based, cross‐sectional study was conducted to clarify the relationship between GSD and the key components of metabolic syndrome and to identify the most efficient predictor of gallstones among different anthropometric indicators of adiposity in Taiwan, as well as to investigate the role of exogenous hormone use, parity, and cirrhosis in the development of GSD.

## 2 MATERIALS AND METHODS

Participant recruitment and sample preservation were performed in the Northeastern Taiwan Community Medicine Research Cohort (NTCMRC, ClinicalTrials.gov, Identifier: NCT04839796).

This community‐based, cross‐sectional study was conducted from April 2014 to August 2017 in 4 districts (Wan‐li, Gong‐liao, Rul‐fan, and An‐le) of northeastern Taiwan. Three districts were rural townships on the northeastern seaboard, and one district was an urban region. Participants aged ≥30 years were recruited from the community through public service announcements, talks among community groups, and clinical notices. Written informed consent was obtained from all the participants. This study conformed to the ethical guidelines of the Declaration of Helsinki and was approved by the Ethical Committee of the Keelung Chang Gung Memorial Hospital.

All participants underwent comprehensive health examinations, where the following data were collected: socioeconomic status; educational level; alcohol, tea, and coffee consumption; smoking history; physical activity; family history; and personal medical histories were obtained through a structured in‐person interview administered by trained nurses, research assistants, and medical students. The patients' medical history of diabetes (DM), hypertension, cerebrovascular disease, cancer, viral hepatitis, asthma, gastritis, renal disease, mental disease, endocrine disease, current use of hormone replacement therapy (HRT) or oral contraceptives (OC), gout, and other conditions was collected. The patients' level of daily physical activity was coded as inactive, moderately inactive, moderately active, or active. An inactive or moderately inactive level of physical activity indicated a sedentary lifestyle. Blood pressure, height, weight, and WC were recorded with the participants wearing light clothes and no shoes. WHtR was calculated by dividing WC in centimeters by height in centimeters, and 0.5 was used as the cutoff value.^15^ BMI‐based definitions for obesity by the Department of Health in Taiwan were used; specifically, the participants were coded as being of normal weight (18.5 kg/m^2^ ≤ BMI < 24 kg/m^2^), overweight (24 kg/m^2^ ≤ BMI < 27 kg/m^2^), and obese (BMI ≥ 27 kg/m^2^). All participants were asked to fast overnight (≥8 h) before blood sample collection. Through blood tests, we gathered data on liver biochemistry; lipid profile; and levels of glucose, insulin, calcium, high‐sensitivity C‐reactive protein, ferritin, uric acid, hepatitis B surface antigen, and anti‐hepatitis C virus.

Alcohol consumption was measured using the Alcohol Use Disorders Identification Test (AUDIT).^16^ This test was administered during an in‐person interview. Participants with a score of ≥8 points on the AUDIT were considered to have heavy alcohol consumption. Participants with a history of alcohol consumption but no excess alcohol intake were considered to have normal alcohol consumption (light‐to‐moderate alcohol intake).

Because insulin resistance (IR) is a key factor in metabolic syndrome, we examined IR using the homeostatic model assessment of IR (HOMA‐IR). Homeostatic model assessment of IR was calculated using the following formula:

HOMA‐IR = fasting plasma insulin (μU/L) × fasting plasma glucose (mmol/L)/22.5

We used 1.4, 2.0, and 2.5 as cut‐off values for HOMA‐IR.^17^, ^18^ The American Diabetes Association criteria for the diagnosis of prediabetes and diabetes, based on glycated hemoglobin (HbA1c) levels, were used as cut‐off values^19^

All abdominal ultrasonography (US) examinations were conducted by an experienced gastroenterologist using the same equipment, and the examiner was blinded to the objectives of the study. The diagnosis of GSD was established based on US findings using a 3.5 MHz transducer. Gallstones were considered to be present when a movable and high‐density intraluminal echogenic material with an acoustic shadow was observed. Moreover, a history of cholecystectomy was recorded.

A US‐based fibrosis scoring system, which includes examination of the liver surface, liver parenchyma, hepatic vessels, and spleen size, was developed to evaluate the degree of hepatic fibrosis. An US diagnosis of cirrhosis was indicated if the score was ≥8. This score had a sensitivity of 45% and a specificity of 93% for the diagnosis of liver cirrhosis.^20^

The Fibrosis‐4 (FIB‐4) index, which was calculated using a noninvasive scoring system, was used to predict liver fibrosis. The FIB‐4 index was calculated using the following formula:

age (years) × aspartate aminotransferase level (U/L]/(platelets [10^9^/L] × [alanine transaminase level [U/L])1/2.

An FIB‐4 index score of <1.45 had a negative predictive value of 90% for advanced fibrosis, which includes early bridging fibrosis to cirrhosis, as determined by the Ishak fibrosis score.^21^

The degree of hepatic steatosis was graded as none, mild, moderate, or severe, based on the discrepancies in echogenicity between the liver and kidneys, degree of posterior attenuation, and visibility of vessels. After the US data were obtained, the presence of cirrhosis and fatty liver was recorded.^22^

According to the National Cholesterol Education Program Adult Treatment Panel III criteria^23^ and the modified Asian criteria for WC,^24^ metabolic syndrome was defined as the presence of any 3 of the following 5 factors: 1) an increased WC (≥90 cm for men and ≥80 cm for women); 2) an elevated triglyceride (TG) level (≥1.695 mmol/L) or the use of TG‐lowering drugs; 3) a low high‐density lipoprotein (HDL) cholesterol level (<1.036 mmol/L in men and <1.295 mmol/L in women) or the use of HDL‐raising drugs; 4) an elevated blood pressure (systolic ≥130 mmHg or diastolic ≥85 mmHg) or the use of antihypertensive drugs; and 5) an elevated fasting glucose level (>5.55 mmol/L) or the use of DM drug treatment.

### 2.1 Statistical analysis

Continuous variables were expressed as mean ± standard deviation. Significant differences between the groups were determined using *t*‐tests and Pearson's chi‐square tests for continuous and categorical variables, respectively. We performed unadjusted and multivariate‐adjusted logistic regression analyses to investigate independent risk factors for GSD. Data processing and analyses were performed using SPSS version 26 (IBM Corp., Armonk, NY, USA). The odds ratio (OR), 95% confidence interval (CI), and *p* values were calculated. Statistical significance was set at *p* value < 0.05. The datasets generated and analyzed in the present study are available from the corresponding author upon reasonable request.

### 2.2 Ethics approval

The study was approved by the ethics committee of the Keelung Chang Gung Memorial Hospital (IRB No: 201800268B0C601).

## 3 RESULTS

Of the participants, 2460 underwent US, of whom 81 (3.3%) had undergone a cholecystectomy. After excluding those who underwent a cholecystectomy and those with incomplete or inadequate data, 2263 participants (788 men and 1475 women) were included. Participants were divided into two groups according to the presence or absence of gallstones. Their characteristics are summarized in Tables 1 and 2.

**TABLE 1:** Sociodemographic characteristics and data of risk behavior among patients with and without gallstones

Variables	Without gallstones (*N* = 2064)	With gallstones (*N* = 199)	*p* Value
**Age, y (mean ± SD)**	55.6(±12.467)	59.2(±10.67)	<0.001
<39	267(12.9%)	11(5.5%)	
40–59	358(17.3%)	22(11.1%)	
50–59	628(30.4%)	63(31.7%)	
>60	811(39.3%)	103(51.8%)	
**Gender(male)**	706(34.2%)	82(41.2%)	0.048
**Tobacco smoking**			0.039
Never	1576(76.4%)	136(68.3%)	
<20 pack year	287(13.9%)	36(18.1%)	
≥20 pack year	201(9.7%)	27(13.6%)	
**Alcohol consumption**			0.494
Non‐drinker	1134(55.2%)	110(55.6%)	
Regular drinker	510(24.8%)	43(21.7%)	
Heavy drinker	411(20.0%)	45(22.7%)	
**Family history of liver disease**	182(8.8%)	12(6.0%)	0.664
**Life style**			0.372
Sedentary	1097(53.3%)	99(50%)	
Mod/vigorous	960(46.7%)	99(50.0%)	
**Exercise** (at least 1 day/week)	1404(68.0%)	150(75.4%)	0.03
**Coffee (cup/day)**			0.555
0	851(42.3%)	84(42.9%)	
1–2	1134(56.3%)	107(54.6%)	
3–4	21(1.0%)	3(1.5%)	
≥5	8(0.4%)	2(1%)	
**Tea**			0.305
<1 day/week	1200(58.3)	108(54.5%)	
≥1 day/week	858(41.7%)	90(45.5%)	
**Lives in rural area**	738(35.8%)	82(41.2%)	0.064
**Education level**			0.008
High‐school degree or lower	1537(74.5%)	165(82.9%)	
College degree or higher	527(25.5%)	34(17.1%)	
**Parity** ^a^	603(47.5%)	69(61.6%)	0.004
**Exogenous hormone drug use** ^b^	32(1.6%)	8(4.1%)	0.021

^a^ Childbirth ≥3 in women.

^b^ Contraceptive or HRT.

**TABLE 2:** Clinical characteristics and laboratory data of patients with and without gallstones

	Without gallstones (*N* = 2064)	With gallstones (*N* = 199)	*p* Value
Continuous parameter (mean ± SD)			
Weight, kg	62.7(±12.24)	64.5(±11.40)	0.044
Body mass index (BMI), kg/m^2^	24.7(±3.84)	25.4(±3.43)	0.01
Waist circumference, cm	80.5(±10.22)	82.9(±9.33)	0.002
Waist‐to‐height ratio	0.51(±0.062	0.52(±0.055)	0.001
SBP, mmHg	129.9(±18.92)	131.9(±18.85)	0.166
DBP, mmHg	78.1(±11.89)	78.5(±11.34)	0.624
Hemoglobin, g/L	138(±15.0)	138.0(±15.7)	0.89
Platelet, 10^9^/L	254.5(±59.07)	251.5(±64.74)	0.488
TC, mmol/L	5.45(±0.983)	5.43(±1.056)	0.852
LDL‐C, mmol/L	3.23(±0.84)	3.22(±0.86)	0.957
HDL‐C, mmol/L	1.48(±0.379)	1.41(±0.414)	0.012
TG, mmol/L	1.374(±1.10)	1.565(±1.485)	0.023
AST, U/L	25.1(±29.39)	26.24(±12.79)	0.595
ALT, U/L	25.3(±19.52)	27.3(±21.39)	0.163
ALP, U/L	65.9(±22.11)	67.9(±18.83)	0.22
Total bilirubin, μmol/L	14.36(±5.30)	14.71(±5.64)	0.436
FPG, mmol/L	5.66(±1.498)	6.04(±1.686)	0.002
HbA1c, %	5.8(±0.80)	6.0(±0.90)	0.005
Uric acid, mmol/L	0.326(±0.082)	0.413(0.080)	0.396
HOMA‐IR	2.15(±3.174)	2.47(±3.026)	0.178
Ferritin, pmol/L	367.6(±360.06)	413(±325.5)	0.171
Calcium, mmol/L	2.38(±0.08)	2.34(0.09)	0.923
Creatinine, μmol/L	66.3(±24.75)	69.84(±23.87)	0.095
FIB‐4 score	1.22(±0.77)	1.37(0.75)	0.009
WBC count(log)	1.748(±0.271)	1.780(±0.278)	0.108
WBC count(10^9^/L)	5.96(±1.753)	6.18(±1.897)	0.104
Insulin(log)	1.583(±0.269)	1.619(±0.263)	0.073
Insulin(pmol/L)	47.60(±43.35)	51.37(±46.96)	0.245
Hs‐CRP (log)	2.0049(±0.46)	2.0683(±0.42)	0.074
Hs‐CRP (nmol/L)	203.79(±431.13)	192.12(±243.43)	0.72
Urine albumin to creatinine(log)	−0.5(±0.506)	0.0053(±0.549)	0.147
Urine albumin to creatinine ratio(mg/mmol)	3.44(±17.39)	5.46(±22.95)	0.133
Categorical parameter			
Platelet(<150 × 10^9^/L)	42(2.0%)	11(5.5%)	0.002
HOMA‐IR≥2.5	473(22.9%)	56(28.1%)	0.096
HOMA‐IR≥2	663(32.1%)	85(42.7%)	0.002
HOMA‐IR≥1.4	1107(53.6%)	124(62.3%)	0.019
Hypertension	491(23.8%)	51(25.6%)	0.561
Plasma glucose level			<0.001
Normal fasting glucose	1345(65.2%)	106(53.3%)	
Impaired fasting glucose (IFG)	440(21.3%)	47(23.6%)	
Diabetes Mellitus^a^	279(13.5%)	46(23.1%)	
HbA1c			0.001
<5.7%	1296(62.8%)	102(51.3%)	
5.7%–6.5%	586(28.4%)	67(33.7%)	
>6.5%	182(8.8%)	30(15.1%)	
Total cholesterol >5.18 mmol/L	1220(59.1%)	116(58.3%)	0.823
LDL >130 mg/dl	863(41.8%)	84(42.2%)	0.913
Non‐HDL cholesterol (>2.59 mmol/L)	1970(95.4%)	182(91.5%)	0.013
Non‐HDL cholesterol (>3.367 mmol/L)	8.2(38.9%)	91(45.7%)	0.058
Chronic kidney disease	324(15.7%)	36(18.1%)	0.378
Fatty liver			0.086
None	894(43.3%)	73(36.7%)	
Mild	616(29.8%)	59(29.6%)	
Moderate or severe	554(26.8%)	67(33.7%)	
FIB‐4(≥3.25)	24(1.2%)	4(2.0%)	0.302
FIB‐4(≤1.45)	1536(74.8%)	128(64.3%)	0.002
Cirrhosis	8(0.4%)	1(0.5%)	0.81
HBV	256(12.4%)	28(14.1%)	0.498
HCV	57(2.8%)	6(3.0%)	0.836
Metabolic syndrome			
Large waist^b^	693(33.6%)	83(41.7%)	0.021
Increased blood pressure^c^	1049(50.8%)	88(44.2%)	0.075
Elevated fasting plasma glucose^d^	718(34.8%)	91(45.7%)	0.002
Elevated triglyceride level^e^	485(23.5%)	64(32.2%)	0.006
Low HDL^f^	470(22.8%)	61(30.7%)	0.012
Factors of metabolic syndrome			0.002
0	521(25.2%)	28(14.1%)	
1	528(25.6%)	47(23.6)	
2	466(22.6%)	53(26.6%)	
3	321(15.6%)	35(17.6%)	
4	182(8.8%)	28(14.1%)	
5	46(2.2%)	8(4.0%)	
Metabolic syndrome^g^	549(26.6%)	71(35.7%)	0.008
BMI (kg/m^2^)			0.007
<24	962(46.6%)	78(39.2%)	
24–27	613(29.7%)	54(27.1%)	
≥27	489(23.7%)	67(33.7%)	
Waist‐to‐height ratio >0.5	1052(51%)	134(67.3%)	<0.001

Abbreviations: ALP, alkaline phosphatase; ALT, alanine aminotransferase; AST, aspartate aminotransferase; DBP, diastolic blood pressure; FPG, fasting plasma glucose; HBV, hepatitis B virus; HCV, hepatitis C virus; LDL, low‐density lipoprotein; SBP, systolic blood pressure; TC, total cholesterol; TG. triglycerides; WBC, white blood cell count.

^a^ 1.HbA1c > 6.5 or fasting sugar >126 mg/dl.

^b^ Waist >90 cm in male or >80 cm in female.

^c^ blood pressure >130 mmHg/85 mmHg.

^d^ Fasting plasma glucose >5.55 mmol/L.

^e^ Triglyceride level >1.695 mmol/L.

^f^ HDL<1.036 mmol/L in male or < 1.295 mmol/L in female.

^g^ Definition according to the National Cholesterol Education Program—Third Adult Treatment Panel (NCEP ATP III).

**FIGURE 1:**
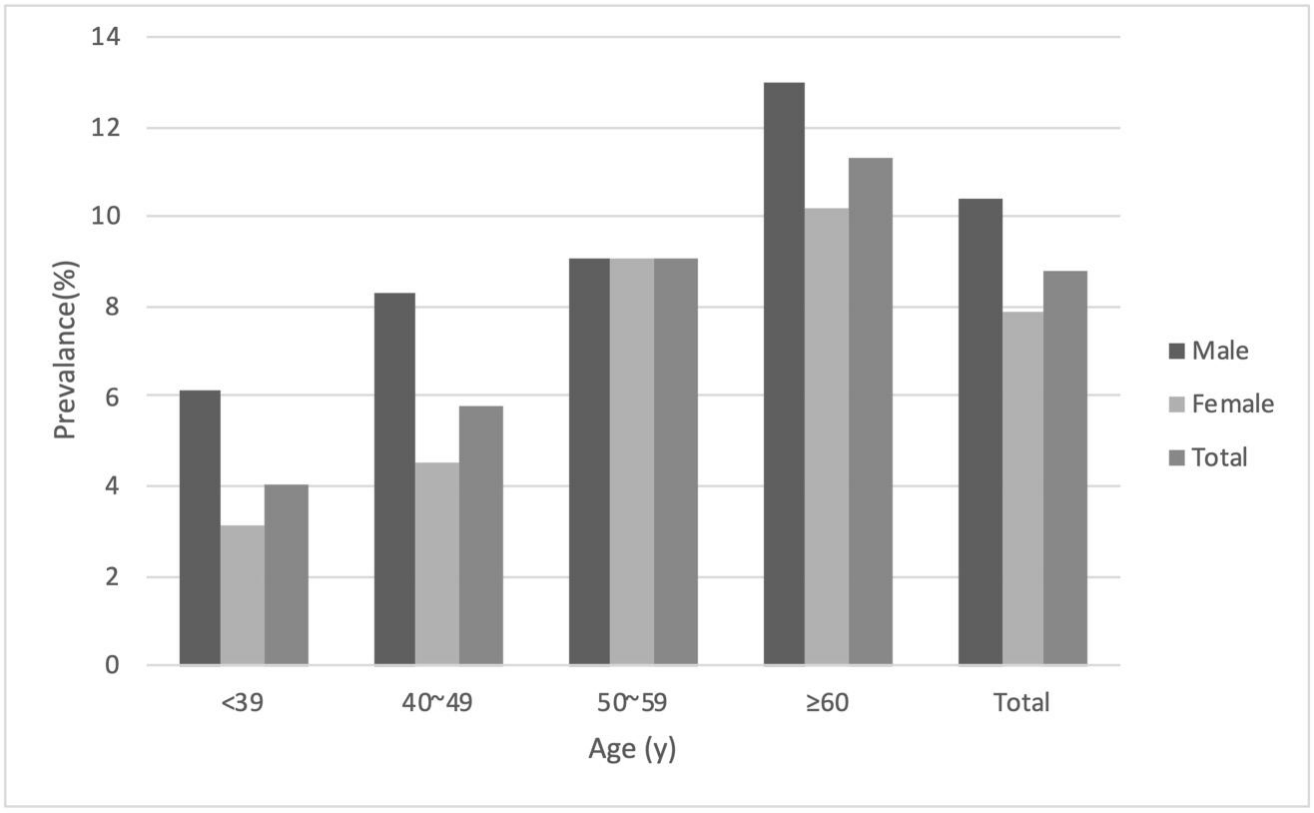
Prevalence of gallstones according to sex and age

The prevalence of gallstones was 8.8%, which was higher in male than in female participants (10.4% vs. 7.9%, *p* = 0.048). The prevalence of GSD increased with age, individuals aged >60 years having the highest prevalence rate (11.3%; Figure 1).

Compared with those without GSD, participants with GSD tended to smoke more, had lower educational qualifications, and exercised less. Compared to those without gallstones, the female participants with gallstones had a history of hormone drug use (4.1% vs. 1.6%, *p* = 0.021) and multiparity (childbirth ≥3; 61.6% vs. 47.5%, *p* = 0.004) (Table 1).

In terms of clinical and laboratory characteristics, patients with GSD had significantly higher BMI, WC, WHtR, fasting glucose, HbA1c, HDL cholesterol levels, and FIB‐4 scores than those without GSD (all *p* < 0.05). Moreover, compared to those without GSD, patients with gallstones had a higher prevalence of DM (14.2% vs. 7.9%, *p* < 0.001) and metabolic syndrome (35.7% vs. 26.6%, *p* = 0.008) (Table 2).

In a univariate analysis, age, male sex, obesity (BMI >27 kg/m^2^), central obesity (increased WC or WHtR >0.5), elevated TG levels, fasting glucose levels of >5.55 mmol/L or the use of glucose‐lowering drugs, low HDL levels or use of HDL cholesterol‐raising drugs, platelet levels <15 × 10^9^/L, HOMA‐IR ≥2, HbA1c >5.7%, DM, FIB‐4 score >1.45, metabolic syndrome, smoking more than 20 packs per year, and multiparity and exogenous hormone drug use (in female participants) were significantly associated with GSD (Table 3). Sex‐stratified univariate analysis showed that WHtR and WHtR >0.5 were significant factors associated with GSD in both sexes.

**TABLE 3:** Univariate analysis of risk factors for gallstone

	Total population		Male		Female	
Variable	OR (95%CI)	*p* Value	OR (95%CI)	*p* Value	OR (95%CI)	*p* Value
Sex(men = ref)	0.742(0.551–0.998)	0.048				
Age	1.024(1.012–1.037)	<0.001	1.018 (1.099~1.037)	<0.001	1.027 (1.011~1.044)	0.001
BMI (kg/m2)	1.049(1.011–1.088)	0.011	1.053 (0.99~1.12)	0.1	1.04 (0.992~1.089)	0.1
Increased BMI (kg/m2)						
24–27 versus < 24	1.086(0.757–1.560)	0.653	0.957 (0.529~1.731)	0.885	1.128 (0.712~1.787)	0.61
≥27 versus < 24	1.69(1.198–2.384)	0.003	1.754 (1.014~3.036)	0.045	1.517 (0.962~2.392)	0.07
Waist circumference (WC), cm	1.022(1.008–1.0370	0.002	1.020 (0.995~1.045)	0.112	1.02 (1.0~1.04)	0.049
Increased WC, cm (*M* ≥ 90; *F* ≥ 80)	1.444(1.073–1.944)	0.015	1.465 (0.911~2.356)	0.115	1.466 (1.0~2.151)	0.05
Waist‐to‐height ratio	39.081(2.99–382.91)	0.002	97.9 (1.764~5433.469)	0.025	19.524 (1.149~331.805)	0.04
Increased waist‐to‐height ratio (≥0.5)	1.983(1.457–2.7)	<0.001	1.67 (1.011~2.742)	0.045	2.117 (1.437~3.14)	<0.001
BP > 130 mmHg/85 mmHg or drug treatment for hypertension	1.305(0.969–1.757)	0.079	1.370 (0.836~2.246)	0.212	1.204 (0.824~1.759)	0.34
Platelet (<15 × 10^9^/L)	2.817(1.426–5.563)	0.003	2.347 (0.988~5.574)	0.053	3.169 (1.135~9.709)	0.043
TC (≥5.18 mmol/L)	0.967(0.720–1.299)	0.823	0.916 (0.580~1.448)	0.708	1.051 (0.711~1.055)	0.802
LDL‐C (>3.367 mmol/L)	1.017(0.757–1.365)	0.913	0.832 (0.518~1.337)	0.448	1.171 (0.801~1.71)	0.415
HDL‐C (<1.036 mmol/L in male or < 1.295 mmol/L in female) or treatment for low HDL‐C	1.475(1.088–2.001)	0.012	1.56 (0.969~2.509)	0.067	1.414 (0.95~2.105)	0.088
TG (≥1.695 mmol/L) or treatment for high triglycerides	1.500(1.107–2.033)	0.009	1.169 (0.733~1.866)	0.511	1.706 (1.141~2.55)	0.009
Non‐HDL cholesterol≥3.367 mmol/L	1.263(0.896–1.780)	0.183	1.163 (0.671~2.016)	0.591	1.299 (0.836~2.018)	0.244
Diabetes mellitus	1.924(1.352–2.738)	<0.001	2.419 (1.428~4.096)	0.001	1.591 (0.983~2.577)	0.059
Fasting glucose						
≥5.55 mmol/L or drug treatment for elevated glucose	1.595(1.191–2.137)	0.002	1.39 (0.879~2.199)	0.159	1.701 (1.163~2.489)	0.006
5.55–6.99 mmol/L versus < 5.55 mmol/L	1.315(0.094–1.843)	0.112	0.902 (0.513~1.587)	0.72	1.626 (1.066~2.48)	0.024
≥6.99 mmol/L versus < 5.55 mmol/L	2.743(1.804–4.172)	<0.001	3.488 (1.92~6.338)	<0.001	2.018 (1.099~3.744)	0.024
HbA1c (%)						
5.7–6.5 versus < 5.7	1.453(1.051–2.007)	0.024	0.955 (0.559~1.632)	0.867	1.866 (1.239~2.809)	0.003
>6.5 versus < 5.7	2.094(1.355–3.238)	0.001	2.174 (1.127~4.124)	0.017	1.961 (1.076~3.576)	0.028
Metabolic syndrome (ATP III)^a^	1.456(1.081–1.962)	0.014	1.153 (0.721~1.844)	0.552	1.666 (1.132~2.452)	0.01
Factors of metabolic syndrome (per 1 factor increased)	1.219(1.106–1.344)	<0.001	1.206 (1.027~1.417)	0.023	1.215 (1.073~1.376)	0.002
HOMA‐IR						
1.4–2 versus < 1.4	1.121(0.749–1.677)	0.579	0.901 (0.457~1.778)	0.764	1.268 (0.767~2.0940)	0.355
>2 versus < 1.4	1.636(1.181–2.266)	0.003	1.662 (1.004~2.753)	0.048	1.574 (1.025~2.416)	0.038
Ferritin ≤67.4157 pmol/L	1.314(0.822–2.101)	0.255	0.449 (0.164~1.230)	0.119	1.473 (0.855~2.54)	0.163
Calcium≥2.5 mmol/L	1.334(0.563–3.160)	0.512	0.610 (0.079~4.701)	0.635	1.792 (0.686~4.682)	0.234
FIB‐4 >1.45(ref.≤1.45)	1.614(1.118–2.192)	0.002	1.769 (1.114~2.811)	0.016	1.42 (0.934~2.159)	0.101
Tobacco smoking						
<20 pack‐year versus never smoke	1.454(0.986–2.143)	0.059	1.401 (0.805~2.483)	0.233	1.331 (0.672~2.636)	0.412
≥20 pack‐year versus never smoke	1.557(1.004–2.412)	0.048	1.475 (0.847~2.57)	0.170	0.911 (0.118~7.035)	0.929
Alcohol consumption						
Regular drinker versus non‐drinker	0.869(0.602–1.255)	0.455	0.949 (0.519~1.734)	0.864	0.779 (0.482~1.259)	0.308
Heavy drinker versus non‐drinker	1.129(0.784–1.626)	0.515	1.268 (0.747~2.54)	0.379	0.720 (0.375~1.381)	0.323
Use of tea (ref. < 1 day/week)	1.166(0.869–1.563)	0.306	1.484 (0.93~2.367)	0.098	0.928 (0.626~1.375)	0.709
Multiparity (Procreation≥3)					1.775 (1.194~2.638)	0.005
Exogenous hormone drug use					2.548 (1.033~2.686)	0.042

^a^ Definition according to the National Cholesterol Education Program—Third Adult Treatment Panel (NCEP ATP III).

Among the participants, WHtR >0.5 was the only significant risk factor for GSD in the multivariate model (OR = 1.65, 95% CI = 1.10–2.48, *p* = 0.017). Furthermore, DM (OR = 1.44, 95% CI = 0.98–2.11, *p* = 0.061) and tobacco smoking (OR = 1.54, 95% CI = 0.99–2.41, *p* = 0.055) was marginally significantly associated with GSD. Age, sex, BMI, low HDL, elevated TG, and FIB‐4 score >1.45 were not significantly associated with GSD (Table 4).

**TABLE 4:** Multivariate analysis of risk factors for gallstone

	Overall		Men		Women	
Variable	OR (95% CI)	*p* Value	OR (95% CI)	*p* Value	OR (95% CI)	*p* Value
Age	1.012(0.996–1.028)	0.141	1.001(0.976–1.027)	0.910	1.015(0.992–1.039)	0.213
Gender male versus women	1.013(0.695–1.475)	0.947				
Body mass index (BMI)	0.990(0.941–1.042)	0.707	1.025(0.943–1.115)	0.561	0.969(0.905–1.037)	0.356
Diabetes mellitus	1.440(0.984–2.108)	0.061	2.062(1.165–3.650)	0.013	1.145(0.672–1.952)	0.618
Low HDL or drug for low HDL	1.206(0.843–1.727)	0.305	1.403(0.801–2.456)	0.236	1.094(0.671–1.783)	0.719
Elevated plasma triglyceride level or drug for elevated TG	1.137(0.793–1.632)	0.485	0.837(0.482–1.456)	0.529	1.263(0.766–2.081)	0.360
Waist‐to‐height ratio >0.5	1.647(1.095–2.476)	0.017	1.285(0.676–2.442)	0.444	1.761(1.027–3.020)	0.040
Smoke<20packyear versus non‐smoker	1.544(0.991–2.406)	0.055	1.487(0.845–2.618)	0.169	1.494(0.680–3.284)	0.318
Smoke>20packyear versus non‐smoker	1.338(0.796–2.248)	0.271	1.350(0.767–2.377)	0.298	1.274(0.158–10.259)	0.820
FIB‐4 score >1.45	1.266(0.865–1.854)	0.225	1.715(0.954–3.082)	0.072	1.039(0.612–1.762)	0.888
Parity^a^					1.305(0.827–2.049)	0.254
Current hormone drug use					2.725(1.086–6.839)	0.033

^a^ Childbirth≥3 in women.

A further sex‐stratified analysis of correlations indicated that DM was the main risk factor for GSD in men (OR = 2.06, 95% CI = 1.17–3.65, *p* = 0.013). Among women, WHtR >0.5 (OR = 1.76, 95% CI = 1.03–3.02, *p* = 0.040) and exogenous hormone intake (OR = 2.73, 95% CI = 1.09–6.84, *p* = 0.033) were significantly associated with GSD. Parity (childbirth ≥3) was not significantly associated with GSD (OR = 1.31, 95% CI = 0.83–2.05, *p* = 0.254).

## 4 DISCUSSION

This community‐based, cross‐sectional study investigated the prevalence of GSD in northeastern Taiwan, determined its independent risk factors, and focused on different indicators of obesity for the prediction of GSD.

We found that central obesity, measured using WHtR, was the most crucial risk factor for GSD in women. Moreover, DM and exogenous hormone intake in men and women, respectively, were positively associated with GSD.

The prevalence of GSD differs depending on ethnicity and geographical location; it was reported to be 20% among Caucasian adults in developed countries, 50.9% among native American people, 4.5% among Japanese people, 3.4% in Korean people, and approximately 7% among Chinese people.^12^ Studies conducted across different geographical locations and ethnicities have demonstrated that the increased prevalence of GSD is etiologically related to dietary factors.^25^ Some studies have indicated that westernized nutrition is associated with an increased prevalence of cholesterol gallstones in Native Americans, Europeans, and urban residents in East Asia.^3^ Due to the westernization of dietary habits and lifestyle, the prevalence of gallstones in Taiwan has exhibited an upward trend in recent years.^26^

The prevalence of GSD in our study was 8.8%, which was lower than that in Western countries (10%–20%),^27^ slightly higher than that in other countries in East Asia,^12^ and similar to that reported in previous studies in Taiwan (5%–11%).^1^ This difference among various regions and ethnic groups might be explained by the various mechanisms of stone formation, and differences in dietary habits and lifestyles between Western and Eastern Asian populations.

The prevalence of gallstones increases with increasing age. In our study, the prevalence of GSD was 11.3% in individuals aged >60 years and only 4% in those aged <40 years. Age is an independent risk factor for GSD, an acquired disease caused by long‐term exposure to environmental risk factors in conjunction with aging. In addition, in older individuals, increased amounts of cholesterol are secreted by the liver and the catabolism of cholesterol to bile acid is decreased.^28^

The role of sex distribution in GSD development remains controversial.^12^ Western studies have indicated that women are generally more susceptible to gallstones than men.^29^ Sex hormones are most likely responsible for the sex‐related differences in the prevalence of GSD. Because of the correlation between female hormones and cholesterol metabolites, some studies have indicated that the higher risk is attributable to female sex hormones, HRT, and OC use.^30^ However, epidemiological investigations have found no evidence for these hypotheses, indicating that the correlation between gallstones and sex may be affected by geographical region and ethnicity. Most studies reporting that female sex contributes to the risk of GSD have been conducted in Western countries,^31^ where the main proportion of gallstones are cholesterol stones. However, studies conducted in Taiwan and other East Asian populations have failed to identify a female predominance in the prevalence of GSD, where a higher proportion of pigment stones were observed.^32^ This difference among various regions and ethnic groups might be explained by differences in the mechanisms of stone formation, composition of gallstones, and dietary habits and lifestyles between Western and East Asian populations. In our study, GSD was more prevalent in the men than in the women. This finding could be attributed to the older age of the male participants. However, sex was not a significant risk factor for GSD in our multivariate analysis.

The sex hormone, estrogen, can result in the hypomotility of the gallbladder, enhance hepatic lipoprotein uptake, inhibit its catabolism to bile salts, reduce bile salt secretion, and promote hepatic secretion of cholesterol into the gallbladder, thus causing cholesterol supersaturation in bile and explaining why more women than men have GSD in occidental areas. Progesterone acts synergistically by impairing gallbladder mobility, which in turn leads to bile stasis. Hormone replacement therapy is associated with an increased risk of GSD in postmenopausal women.^14^ Some studies have reported a relationship between OC use and a high prevalence of GSD.^33^ A meta‐analysis including 556,620 participants indicated that HRT was positively associated with GSD, and OC use was not a risk factor for cholelithiasis.^34^ In our study, HRT and OC use were independent risk factors for GSD in women. Considering the mean age of the female participants in this study, we presumed that most participants with exogenous hormone drug use in our study were postmenopausal HRT users, rather than OC users.

However, the association between parity and GSD remains controversial.^35^ Because incomplete gallbladder emptying occurs in late pregnancy and cholesterol saturation in bile may be increased during pregnancy, multiparity is considered to be a major factor for cholesterol gallstone formation in women^13^ a view that has been supported by several studies. However, two large studies, one from Germany and the other from France, found no such associations.^36^ The present study indicated that multiparity was associated with GSD in the univariate analysis; however, parity was not a significant risk factor in the multivariate logistic regression analysis. Parity increases with age, which is a crucial factor in GSD, and the difference in stone composition may explain this result.

The association between gallstones and metabolic syndrome is well documented.^37^ Four pathophysiological processes underlie metabolic syndrome: IR, central obesity, lipid level, and blood pressure.^38^ An association between metabolic syndrome and GSD was observed, and we found that among metabolic syndrome components, lower HDL, elevated TG, elevated fasting glucose, and increased WC were significantly correlated with GSD prevalence in our univariate logistic regression. Moreover, DM and central obesity, defined as a WHtR ≥0.5, were strong risk factors for GSD.

Central obesity is a risk factor of GSD.^39^ The association between obesity and GSD is attributable to the increased hepatic secretion of cholesterol with the resultant cholesterol‐supersaturated bile that precipitates as cholesterol gallstones. In addition, some studies have reported that obese patients have impaired gallbladder motility, which is mediated by decreased sensitivity to cholecystokinin.^5^

Body mass index, WC, WHR, and WHtR are the most widely used anthropometric proxy indicators for obesity.^40^ Several studies have used anthropometric measures to evaluate the association between obesity and gallstones and have indicated that BMI, WC, WHR, and WHtR are risk factors for GSD.^41^ However, two cohort studies have reported that central obesity measured by WC or WHR was associated with symptomatic gallstones, independent of BMI.^42^ Therefore, the strongest risk factor among the anthropometric indicators for gallstones remains inconclusive.

Several underlying mechanisms support the utility and accuracy of the WHtR in the assessment of central obesity. Body mass index is an indicator of lean body mass and is unrelated to fat distribution. Therefore, BMI cannot be used to distinguish between visceral and subcutaneous fat. Individuals with high visceral fat may have a normal or high BMI. Moreover, BMI cannot be used to distinguish between individuals with excess adipose tissue and those with a high muscle mass. Unlike BMI, WHtR accounts for abdominal fat accumulation, and central obesity is more strongly associated with cardiovascular risk than body weight. Moreover, in a meta‐analysis, WHtR was more strongly correlated with cardiovascular risk factors than with other anthropometric indicators of central obesity, such as BMI.^8^

Waist circumference is a simple indicator of central adiposity used to predict cardiovascular risk, which assumes that individuals with the same WC tend to have a similar cardiovascular risk as those with a different stature. However, this assumption is questionable, because one Mexican study argued that shorter individuals have more visceral fat than taller individuals with the same BMI.^9^ In a radiological study, WHtR was more strongly correlated with visceral fat than WC.^10^ A longitudinal study reported that after height or hip circumference was controlled for, the predictive power of WC for the incidence of hypertension improved,^11^ which might be explained by the fact that individuals with the same WC may have different proportions of waist adiposity, depending on their stature. Furthermore, WHR may remain unchanged even when body size changes because WC and hip circumference may change in the same proportion, whereas WHtR changes only when WC changes, given that height remains constant in most adults.

WHtR has been strongly associated with several chronic diseases, such as hypertension, cardiovascular events, type 2 diabetes, and metabolic syndrome, and has thus been extensively researched.^7^ To our knowledge, only one population‐based, cross‐sectional study has investigated the relationship between the WHtR and GSD and demonstrated that a high WHtR is related to an increased risk of GSD.^12^ Among Taiwanese adults, a WHtR >0.5 is an easily applicable and reliable indicator to assess central adiposity and associated cardiometabolic risk, even among healthy individuals without risk factors for metabolic syndrome.^43^ In our study, 1) increased WHtR, BMI, and WC were significantly associated with GSD in a univariate analysis, and 2) increased WHtR was a more crucial risk factor for GSD than BMI and WC. Furthermore, increased WHtR was a significant risk factor for GSD in the multivariate analysis in women. The pathophysiological mechanism underlying the strong association between WHtR and gallstone risk in women remains unclear. Sex differences have also been reported in previous studies. A cross‐sectional study conducted in Taiwan reported that abdominal obesity is associated with GSD in women, but not in men.^11^ Another study conducted in China revealed that obesity is positively associated with GSD in women, but not in men.^44^ One possible explanation for our result is that women had a shorter stature than older men, and height can reflect environmental exposure in early life. Further studies are needed to confirm this hypothesis.

DM is associated with an increased risk of GSD. A population‐based cohort study reported that diabetes is associated with an increased risk of symptomatic GSD.^45^ The possible pathogenic mechanisms for this increased risk are gallbladder hypomotility–induced biliary stasis due to autonomic neuropathy and increased biliary saturation in DM.^46^ IR may play a crucial role in gallstone formation. In our study, a higher prevalence of GSD was noted in patients with DM than in those without (Table 1). However, consistent with the findings of a previous study^44^ the present study found a positive association between DM and GSD in men, but not in women. Our finding was inconsistent with that of a case‐control study that had a smaller sample size, but demonstrated an increased prevalence of gallstones only in female patients with DM.^47^ The role of sex‐related differences in the association between DM and GSD is complex, and should be examined in future studies.

Cirrhosis is a well‐established risk factor for both pigmented and cholesterol gallstones.^3^ Liver cirrhosis may cause gallbladder hypomotility, bile malabsorption, diminished bile synthesis, elevated enterohepatic cycling of unconjugated bilirubin, and gallstones.^48^ Another passible pathogenetic pathway underlying gallstone formation in liver cirrhosis may be attributed to steatohepatitis, a key etiology of advanced hepatic fibrosis and cirrhosis, and a hepatic component of metabolic disorders representing IR.^49^, ^50^ In the present study, we found that a nonfibrotic liver, as determined using the FIB‐4 score, was negatively associated with GSD in our univariate analysis; however, the significance disappeared when the FIB‐4 score was adjusted with respect to other factors in the multivariate logistic regression. Our results can be partially explained by the relatively small proportion of participants with advanced fibrosis and cirrhosis and the differences in gallstone composition.

Our study had several limitations. Firstly, its retrospective and cross‐sectional design precludes causal inferences. Thus, additional large‐scale, longitudinal cohort studies are required to identify the risk factors for GSD. Secondly, we did not explore the frequency, dose, and duration of drug exposure or their relationship with GSD. Thirdly, because our participants were of East Asian descent, our results could not be generalized to other ethnicities. Fourthly, we could not determine the gallstone composition; therefore, factors related to both types of gallstones may be insignificant. The severity of liver steatosis and fibrosis was assessed using US, and the participants did not undergo transient elastography, which is a more accurate, noninvasive method for the assessment of liver fat and fibrosis. Furthermore, certain recognized gallstone risk factors, such as family history of GSD and changes in body weight, were not included in the present study.

In conclusion, our study demonstrated that GSD is independently associated with central obesity and exogenous hormone intake in women. Among the anthropometric indicators used to assess central obesity, the WHtR was the most accurate predictor of GSD. Gallstone disease was independently associated with DM in men. This community‐based study in Taiwan will aid future cross‐country studies, etiological inferences, and disease prevention.

## AUTHOR CONTRIBUTIONS

Chien and Chou provided the concept and design of the study. Lin, L. W. Chen, Hu, Chang, Yen, S.W. Chen, and Liu collected the data. Chien and Chou performed data analysis and interpretation. Chien and Chou wrote the manuscript. All the authors have read and approved the final manuscript.

## CONFLICT OF INTEREST

The authors declare no conflicts of interest.
